# Oxidative Stress, Gut Bacteria, and Microalgae: A Holistic Approach to Manage Inflammatory Bowel Diseases

**DOI:** 10.3390/antiox14060697

**Published:** 2025-06-09

**Authors:** Shani Shoham, Noam Pintel, Dorit Avni

**Affiliations:** 1 Bio-Compounds and Immune-Mediated Diseases Group, MIGAL—Galilee Research Institute, 1101600 Kiryat Shemona, Israel; shani.sh@migal.org.il (S.S.);; 2 Department of Biotechnology, Faculty of Science and Technology, Tel Hai College, 1220800 Kiryat Shemona, Israel

**Keywords:** inflammatory bowel disease, microalgae, microbiome, oxidative stress, antioxidants, ROS, short-chain fatty acids, antibiotics, microalgae-based compounds

## Abstract

Oxidative stress is a recognized contributor to the pathophysiology of inflammatory bowel disease (IBD), exacerbating chronic inflammation and tissue damage. While traditional IBD therapies primarily focus on immune modulation, alternative approaches that address oxidative stress and promote gut microbial health present new opportunities for symptom relief and disease management. Microalgae, known for their potent antioxidant, anti-inflammatory, and prebiotic properties, show promise in alleviating oxidative damage and supporting beneficial gut bacteria. This review explores the multifaceted role of oxidative stress in IBD and highlights the therapeutic potential of microalgae-derived compounds. In addition, it examines the synergistic benefits of combining microalgal antioxidants with probiotics to promote gut homeostasis. Advances in delivery systems, including nanotechnology and symbiotic bacteria–microalgae interactions, are also discussed as emerging approaches for targeted treatment. The review concludes by identifying future research priorities focused on clinical translation and microalgae-based bioengineering innovations to enhance the efficacy and accessibility of therapeutics for IBD patients.

## 1. Introduction

Inflammatory bowel disease (IBD), comprising Crohn’s disease (CD) and Ulcerative colitis (UC), is a chronic, relapsing inflammatory condition of the gastrointestinal tract that affects millions of individuals globally. Its prevalence continues to rise, particularly in industrialized and urbanizing regions, a trend strongly linked to environmental changes, dietary westernization, and lifestyle factors. Clinically, IBD manifests through symptoms such as chronic diarrhea, abdominal pain, fatigue, rectal bleeding, and weight loss [1,2,3], with complications including intestinal fibrosis, strictures, and an increased risk of colorectal cancer in more advanced cases that may develop [1,2]. These manifestations severely impact quality of life and require long-term pharmacological, nutritional and behavioral interventions, and dietary changes [1,4].

**Figure 1:**
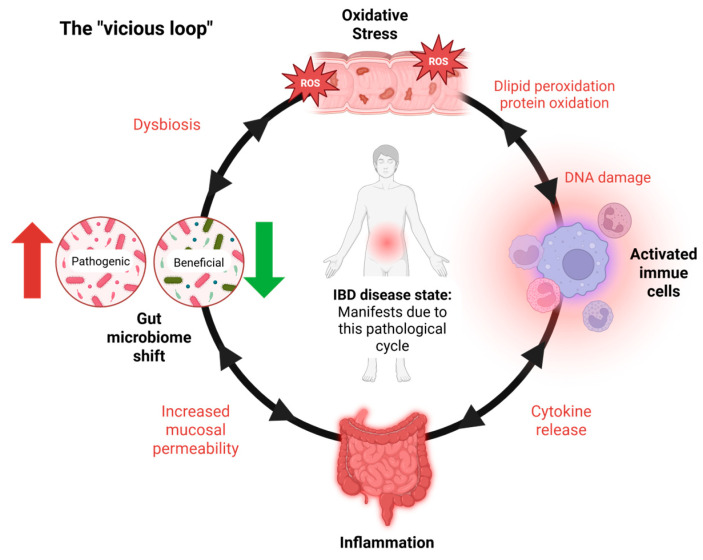
IBD’s vicious loop. The vicious cycle underlying IBD pathogenesis. Inflammatory bowel disease (IBD) is sustained by a self-reinforcing loop of oxidative stress, immune activation, and microbial dysbiosis. The cycle begins with oxidative stress, largely driven by activated immune cells producing ROS. This damages the epithelial barrier, resulting in inflammation and gut microbiota shifts. These changes promote further immune activation and ROS production, maintaining a chronic inflammatory state. IBD symptoms and tissue damage manifest primarily during the inflammatory stage, which is both a result and a driver of the cycle. Notably, the interactions within this loop are bidirectional, with each factor capable of amplifying the others.

IBD primarily involves persistent inflammation of the gastrointestinal (GI) tract, driven by an abnormal immune response to environmental and microbial factors [5]. A central pathological feature of IBD is oxidative stress, which plays a critical role in both the initiation and perpetuation of intestinal inflammation [6,7]. This stress results from an imbalance between the production of reactive oxygen species (ROS) and the body’s antioxidant defense mechanisms [8]. In IBD, activated immune cells such as neutrophils and macrophages produce excessive ROS at inflamed intestinal sites [9], leading to damage to cellular components—including lipids, proteins, and DNA. This oxidative damage compromises the integrity of the intestinal epithelial barrier, increases mucosal permeability, and facilitates the translocation of luminal antigens and pathogens, thereby intensifying the immune response [10,11]. Consistently elevated levels of oxidative markers like malondialdehyde (MDA), along with reduced activity of antioxidant enzymes such as superoxide dismutase (SOD), catalase (CAT), and glutathione peroxidase, further underscore the role of redox imbalance in IBD progression [10,11]. The intestinal epithelium’s heightened vulnerability to oxidative injury exacerbates barrier dysfunction and fuels a self-reinforcing cycle of inflammation and tissue damage, which sustains the chronic nature of IBD [2,3] (Figure 1).

Recent evidence also highlights the gut microbiome’s pivotal role in modulating oxidative stress and intestinal inflammation [12]. In healthy individuals, a balanced and diverse microbial community supports epithelial integrity, immune regulation, and antioxidant capacity. In contrast, IBD is characterized by microbial dysbiosis, marked by reduced diversity, the depletion of beneficial commensals such as *Faecalibacterium prausnitzii*, and the overrepresentation of pathogenic species such as *Escherichia coli* [13]. This dysbiosis contributes to impaired short-chain fatty acid (SCFA) production, increased intestinal permeability, and immune dysregulation [14].

Interestingly, recent multi-omics Mendelian randomization studies have examined the complex interplay between gut microbiota and oxidative stress-related genes in Crohn’s disease. These analyses identified key genetic contributors, including Signal Transducer and Activator of Transcription 3 (*STAT3*), which regulates inflammatory signaling; Mucin 1 (*MUC1*), essential for maintaining mucus barrier integrity and mediating host–microbe interactions; and Protein Kinase AMP-Activated Non-Catalytic Subunit Beta 1 (*PRKAB1*), which plays a critical role in energy homeostasis and redox balance. The findings highlight a mechanistic link among epithelial barrier function, oxidative stress, and inflammatory signaling in shaping microbial–host interactions in Crohn’s disease. Moreover, the study suggests that oxidative stress-related genes may contribute to disease pathogenesis through mechanisms involving DNA methylation, altered gene expression, and microbiome modulation [6].

**IBD’s global economic burden and limitations of current therapies.** Recent epidemiological data indicate that global trends in IBD are evolving significantly, with stark regional variations in hospitalization rates [15]. In Western regions such as North America and Europe, where advanced therapies such as biologics are widely available, hospitalization rates have stabilized or declined due to the transition of care to outpatient settings [15]. Conversely, newly industrialized countries in Asia and Latin America are experiencing rapid increases in hospitalization rates, reflecting rising IBD incidence and limited access to advanced medical therapies [15]. This significantly impacts patients’ quality of life and places a substantial burden on healthcare systems [1,2,10]. IBD carries a considerable economic burden, with direct healthcare expenses—encompassing ambulatory visits, hospitalizations, and medications—averaging between USD 9000 and USD 12,000 per individual annually in high-income regions [1,3,4]. However, these estimates may not fully capture variations in disease severity, healthcare accessibility, and disparities in infrastructure across different regions. In addition to direct medical costs, indirect expenses arising from productivity losses due to absenteeism, presenteeism, and other intangible factors significantly contribute to the overall financial impact [16]. Recent estimates indicate that IBD affects approximately 0.7% of the U.S. population, accounting for 2.39 million individuals [16]. Based on the Cost Commission’s annual average estimates, the total economic burden of IBD in the U.S. is projected to be approximately USD 50 billion annually [16].

Current therapeutic strategies primarily aim to suppress inflammation, induce and maintain remission, and improve patients’ quality of life; however, they fall short of offering a definitive cure. These therapies include anti-inflammatory agents (e.g., corticosteroids, aminosalicylates), which are effective for acute symptom control but are associated with significant adverse effects such as osteoporosis and increased susceptibility to infections [1,10]. Immunomodulators and biologic agents targeting specific inflammatory mediators, such as Tumor Necrosis Factor (TNF)-α and Interleukin (IL)-6, offer targeted suppression but may lose efficacy over time and pose risks of systemic immunosuppression [1,2]. In refractory cases where pharmacological management fails, surgical intervention becomes necessary. While surgery can provide symptomatic relief and improve quality of life, it carries the risk of postoperative complications, including infections, anastomotic leakage, and long-term functional impairments [3,10].

Despite their therapeutic potential, these approaches do not adequately restore mucosal barrier function or resolve underlying gut dysbiosis, and oxidative stress factors are increasingly recognized as central to IBD pathogenesis [2,10].

**Microbiome-targeted and microalgae-based interventions.** Amid these limitations, there is a growing shift toward microbiome-targeted and natural compound-based therapies. Natural agents, including prebiotics, probiotics, and postbiotics, have gained increasing attention for their ability to modulate the gut microbiome, promote epithelial barrier integrity, and modulate inflammatory responses [17]. For instance, probiotic strains such as *Lactobacillus* and *Bifidobacterium* have been shown to upregulate anti-inflammatory cytokines and reinforce tight junction integrity [2,10]. Prebiotics serve as fermentable substrates for beneficial gut microbes, leading to the generation of short-chain fatty acids (SCFAs) that confer mucosal protection and immunomodulatory benefits [10,11].

Targeting oxidative stress has also emerged as a promising therapeutic strategy. Both synthetic and natural antioxidants have demonstrated efficacy in neutralizing ROS and mitigating oxidative tissue damage [2,11]. In this context, microalgae-derived compounds offer significant promise. Microalgae such as *Spirulina* and *Chlorella* are rich sources of bioactive molecules, including phycocyanins and carotenoids, which exhibit potent antioxidant and anti-inflammatory activities [1,2,11]. In addition, microalgal polysaccharides possess prebiotic properties that support the proliferation of beneficial gut microbiota and contribute to the restoration of microbial homeostasis [10,11,18].

IBD progression is marked by a self-perpetuating loop (Figure 1) wherein inflammatory processes stimulate excessive ROS production, resulting in oxidative damage to the intestinal epithelium. This, in turn, compromises epithelial integrity, exacerbates mucosal permeability, and contributes to sustained dysbiosis and immune activation [2,3,10].

Addressing oxidative stress and microbiota imbalance thus represents a dual therapeutic target, and natural compounds from microalgae are well-positioned to fulfill this role [4,10,11].

This review examines the interplay between oxidative stress, gut microbiota dysbiosis, and microalgae-based therapeutic strategies in IBD. By synthesizing recent evidence, we aim to elucidate the potential of microalgae and microbiome-modulating approaches to disrupt the pathogenic cycle, restore gut homeostasis, and improve clinical outcomes in patients with IBD.

## 2. Mechanisms of Oxidative Stress in IBD and Gut Dysfunction

**Disruption of tight junctions by oxidative stress**. In the gut, the mucosal epithelial barrier protects against inflammation and maintains intestinal tract homeostasis by isolating luminal bacteria, toxins, and antigens from the mucosal immune system [19]. Oxidative stress-induced epithelial damage compromises gut barrier integrity, as evidenced by reduced expression of tight junction proteins such as occludin and Zonula Occludens-1 (ZO-1), which are crucial for maintaining intestinal permeability [20]. Disruptions in these proteins facilitate increased gut permeability, allowing luminal antigens and microbes to penetrate the mucosa, triggering chronic inflammation [20]. Recent studies have explored how natural bioactives may reverse this damage. By targeting these disruptions, plant-based polysaccharides have demonstrated efficacy in restoring barrier function and reducing intestinal permeability through modulation of oxidative stress pathways [21]. Wang et al. (2022) [21] investigated the effects of crude *Dendrobium fimbriatum* polysaccharide fraction W1 (cDFPW1), derived from the orchid species *Dendrobium fimbriatum*, in a dextran sodium sulfate (DSS)-induced colitis model. Their study employed histopathological analysis, tight junction protein immunohistochemistry, and intestinal permeability assays to assess the polysaccharide’s protective effects. The results showed that cDFPW1 significantly upregulated occludin and ZO-1 expression in colonic epithelial tissues, improving gut barrier integrity [21]. Additionally, serum biomarkers of permeability, including D-lactic acid and endotoxin levels, were markedly reduced in cDFPW1-treated mice compared to controls [21]. Mechanistically, cDFPW1 was found to activate the nuclear factor erythroid 2-related factor 2 (Nrf2) pathway, responsible for enhancing the antioxidant response, while simultaneously inhibiting nuclear factor-kappa B (NF-κB) activation, which reduced oxidative stress and inflammatory cytokines such as TNF-α and IL-6 [21].

Transcription factors such as NF-κB and Nrf2 regulate pro-inflammatory and antioxidant responses. NF-κB drives cytokine production and inflammatory cascades, while Nrf2 activation enhances antioxidant defenses [21]. In studies involving murine colitis models, the activation of Nrf2 improved epithelial integrity and reduced IBD severity by upregulating antioxidant enzymes such as NAD(P)H quinone oxidoreductase 1 (NQO1), heme oxygenase-1 (HO-1), and glutathione S-transferase (GST) [22].

Specifically, Liu et al. (2022) [23] demonstrated that Nrf2-deficient mice exhibited increased severity of DSS-induced colitis, characterized by elevated MDA levels and reduced glutathione peroxidase (GPx) activity [23]. Conversely, Sahoo et al. (2023) [24] further detailed that Nrf2 activation via phytochemicals like curcumin and resveratrol significantly reduced oxidative stress by increasing SOD and CAT activities [24]. In their experiments, these natural compounds inhibited NF-κB-mediated pathways, resulting in decreased levels of inflammatory mediators such as IL-1β and IL-6, while simultaneously boosting the expression of tight junction proteins such as occludin and ZO-1 [23,24].

Furthermore, oxidative stress-induced epithelial cell damage exacerbates mucosal dysfunction by disrupting the structural integrity of the intestinal barrier [25]. According to Bourgonje et al. (2020) [25], excessive ROS damages cellular macromolecules, such as lipids, proteins, and DNA, and targets cysteine redox switches in key proteins, which regulate cellular signaling pathways. These disruptions impair epithelial cell function and increase intestinal permeability, perpetuating chronic inflammation and tissue injury in IBD [25,26].

**Clinical Markers of Oxidative Stress in IBD**. Clinical studies have identified key biomarkers of oxidative stress that correlate with IBD severity. MDA, a lipid peroxidation product, is significantly elevated in patients with active IBD, reflecting heightened oxidative damage [23,25]. Antioxidant enzyme levels, including SOD and glutathione peroxidase (GPx), are often depleted, indicating compromised antioxidant defenses in the inflamed gut [25,26]. These biomarkers provide insights into disease activity and offer potential for evaluating therapeutic efficacy. For instance, antioxidant therapies that reduce MDA levels and restore SOD activity have demonstrated clinical benefit in managing IBD [24] (See previous sub-chapter).

**Recent Advances in Antioxidant-Related Therapeutic Strategies**. Recent advancements in nanotechnology offer promising therapeutic approaches for mitigating oxidative stress in IBD [23]. Nanoparticles delivering antioxidants such as curcumin, glutathione, or catalase allow targeted delivery to inflamed gut tissues, enhancing therapeutic efficacy and minimizing systemic side effects [23]. These nanocarriers stabilize antioxidants and enable site-specific release, addressing the limitations of conventional antioxidant therapies [23]. Natural compounds such as polyphenols (e.g., resveratrol, curcumin) and flavonoids (e.g., quercetin) exhibit potent ROS-scavenging properties [24]. Preclinical studies have demonstrated that these compounds neutralize oxidative damage and modulate inflammatory pathways, reducing colitis severity in murine models [24,25]. Such strategies highlight the therapeutic potential of combining nanomedicine with natural antioxidants to target oxidative stress in IBD. These approaches, while promising, are part of a broader therapeutic paradigm that also includes microbiome-targeted interventions.

## 3. The Involvement of Gut Microbiome in IBD

The gut microbiome plays an important role in the pathogenesis and progression of IBD, with distinct differences observed between IBD patients and healthy individuals, including reduced microbial diversity, species richness, and stability [13,27]. A balanced microbial community is essential for maintaining intestinal homeostasis, supporting immune regulation, and preserving the integrity of the epithelial barrier. In IBD patients, this balance is often disrupted, a condition known as dysbiosis, and is associated with decreased beneficial bacteria, such as *Faecalibacterium prausnitzii*, and an increased prevalence of pathogenic microbes, including adherent-invasive *Escherichia coli* strains. These microbial alterations lead to an imbalance between pro-inflammatory and anti-inflammatory bacteria, evident even at disease onset [28,29]. These microbial shifts can also lead to increased intestinal permeability, impaired immune tolerance, and heightened inflammatory responses. Moreover, the altered microbiota can influence the production of metabolites such as short-chain fatty acids, which are important for mucosal healing and immune modulation [14].

Genetic susceptibility, environmental influences, and immune responses further interact with the microbiota, perpetuating dysbiosis and inflammation [30,31].

Beyond taxonomic shifts, recent technologies have enabled deeper exploration of the microbiome’s functional roles in IBD. Advanced omics technologies have revolutionized our understanding of gut microbiota in IBD [32]. These techniques, including 16S rRNA sequencing, metagenomics, and metabolomics, provide deeper insights into compositional and functional changes in the gut microbiome of IBD patients [29,32]. Studies have revealed significant perturbations in bacterial community composition, diversity, and associated metabolic pathways in IBD, including decreased abundance of Bacteroidetes and Firmicutes, and increased disease-associated phyla like Proteobacteria [33]. Such shifts are closely tied to disease severity and immune activation, as metabolomic analyses have identified potential biomarkers for differentiating IBD from healthy individuals, such as 6,7,4′-trihydroxy isoflavone and thyroxine 4′-o-.beta.-d-glucuronide, alongside reduced butyrate production and altered short-chain fatty acid metabolism [14,28,34,35]. Specific genera such as *Akkermansia*, *Ruminococcus*, and *Faecalibacterium* have been identified as potential biomarkers for IBD diagnosis and prognosis [14]. Longitudinal studies show that microbial dysbiosis correlates with disease severity and can predict treatment responses, though it often persists even in patients achieving mucosal healing [36]. Microbiome-based biomarkers are increasingly seen as promising tools for improving IBD diagnosis, monitoring, and personalised therapies [36]. Emerging treatments, including probiotics, prebiotics, antibiotics, fecal microbiota transplantation (FMT), and gene manipulation, are being explored to modulate the microbiome and restore its balance [27,31]. These interventions, particularly those involving microalgae-derived prebiotics, will be discussed in the following sections.

## 4. Oxidative Stress and Gut Microbiota Axis

Oxidative stress significantly alters the gut microbiota, contributing to the dysbiosis commonly observed in IBD [12]. Elevated luminal ROS levels impair beneficial microbial species, favoring the growth of pathobionts such as *Escherichia coli* while reducing commensals like *Lactobacillus* and *Bifidobacterium* [25,37]. Furthermore, natural interventions such as cDFPW1 (polysaccharide) demonstrate the capacity to modulate gut microbiota by enhancing beneficial species like *Lactobacillus* and reducing pathogenic taxa, thereby alleviating dysbiosis-associated inflammation [21]. This shift in microbial composition further amplifies inflammation, creating a vicious cycle of oxidative damage and dysbiosis (Figure 1). Studies have shown that mitochondrial dysfunction in epithelial cells exacerbates ROS production, directly impacting microbial diversity [38]. In a colitis model, heightened ROS levels were linked to a decline in microbial richness and increased colonization by pro-inflammatory species, further driving disease progression [37,38]. These findings underscore the critical role of oxidative stress in shaping gut microbial ecosystems during IBD.

At the microbial level, oxidative stress can interfere with the transcriptional and translational machinery of bacteria, impairing gene expression and protein synthesis [39]. To survive such stress, bacteria have evolved protective systems including ROS-scavenging enzymes like CAT and peroxidase, as well as terminal oxidases in their respiratory chains that help mitigate oxidative damage under aerobic conditions [40]. Key redox-sensitive transcription factors, such as Oxidative stress regulator (OxyR) and Peroxide response regulator (PerR), enable bacteria to sense intracellular hydrogen peroxide and activate defense responses, including reducing H_2_O_2_ concentrations, limiting intracellular iron, and repairing oxidative macromolecular damage [41]. These defense mechanisms are vital for bacterial persistence and function in oxidative environments, including inflamed gut niches encountered during IBD [41].

Additionally, SCFAs, particularly acetate, propionate, and butyrate, are crucial metabolites produced by the gut microbiota through dietary fiber fermentation. These compounds play key roles in maintaining gut and systemic health by serving as an energy source for colonic epithelial cells, reducing inflammation via G-protein-coupled receptors’ (GPCRs’) activation, and enhancing the intestinal barrier by stimulating mucus production and tight junction protein expression [42,43]. Additionally, SCFAs lower colonic pH to support beneficial bacteria and act as epigenetic modulators to suppress inflammation. However, oxidative stress and inflammation can significantly impair SCFA production. ROS disrupt the gut microbiota, depleting SCFA-producing bacteria, while chronic inflammation hinders microbial fermentation processes. Moreover, excessive ROS can directly oxidize SCFAs, reducing their availability and efficacy [44]. These disruptions are compounded by intestinal barrier damage caused by ROS and inflammation, which decreases the availability of fermentation substrates. Despite this vulnerability, SCFAs possess protective properties, such as enhancing antioxidant enzyme activity, neutralizing ROS, and inhibiting pro-inflammatory pathways like NF-κB. They also repair oxidative damage to the intestinal barrier by promoting epithelial regeneration and maintaining tight junction integrity [43]. Given their central role in gut homeostasis, supporting SCFA-producing microbes through targeted therapies, such as microalgae-derived prebiotics, may offer a promising therapeutic strategy.

## 5. Microalgae as a Natural Source in Treating IBD

Microalgae are diverse unicellular photosynthetic organisms found in freshwater and marine environments. They are a rich source of bioactive compounds with a wide range of applications in nutrition, pharmaceuticals, and disease treatment [45]. Microalgae’s rapid growth, adaptability to diverse environmental conditions, and high photosynthetic efficiency make them a sustainable and renewable resource [46]. Microalgae have garnered attention as a sustainable and potent source of therapeutic compounds for managing inflammatory bowel disease (IBD). Recent preclinical studies have begun validating these properties in experimental models of gut inflammation.

Species such as *Spirulina platensis*, *Chlorella vulgaris*, and *Phaeodactylum tricornutum* are rich in bioactive compounds, including phycocyanin, polysaccharides, polyphenols, and unsaturated fatty acids, which exhibit anti-inflammatory, antioxidant, and immunomodulatory properties [47]. In a recent study, Zhou et al. (2023) [48] evaluated the effects of pressurized liquid extracts (PLEs) from the abovementioned microalgae on gut health and inflammation. Using in vitro colonic fermentation models, the researchers demonstrated that PLEs significantly reduced the activation of the inflammatory NF-κB pathway, increased beneficial bacterial strains (*Lactobacillus* and *Bifidobacterium*), and inhibited pathogenic bacteria. Additionally, PLEs enhanced SCFA production, including butyrate and propionate, which are critical for maintaining gut barrier integrity [48]. In line with these findings, Omar et al. (2022) [49] used a murine colitis model to show that *Chlorella vulgaris* restored gut microbiota diversity, reduced epithelial damage, and downregulated pro-inflammatory cytokines such as TNF-α and IL-6 [49].

**Microalgae as a source for sustainable bio-compounds.** These unicellular organisms are rich in essential nutrients, including proteins, peptides, polysaccharides, lipids, carotenoids, and phenolic compounds, which provide antioxidant, anti-inflammatory, and other health-promoting properties [50]. Microalgae such as *Spirulina* (*Arthrospira platensis*) and *Chlorella* are particularly noteworthy for their high protein content, reaching up to 70% of their dry weight, surpassing many conventional plant and animal protein sources [47]. This high nutrient density makes them not only viable as therapeutic agents but also as functional foods. These abundant proteins possess a favorable amino acid profile, including essential amino acids such as leucine, lysine, and arginine. Furthermore, bioactive peptides derived from microalgae, such as those isolated from *Spirulina* and *Chlorella*, have demonstrated potential therapeutic effects, including reducing inflammation and oxidative stress by modulating pathways like NF-κB and cytokine production [11,47]. Beyond nutrition, these compounds exert pharmacological effects.

The potential health applications of microalgae extend to chronic conditions. Compounds like phycocyanin, a pigment–protein complex found in *Spirulina*, have shown promise in reducing gut inflammation and oxidative damage [51]. Moreover, various other algae-derived bioactive compounds, including fucoxanthin, polysaccharides, polyphenols, carotenoids, and lipids from micro- and macroalgae, have demonstrated substantial therapeutic potential. These compounds modulate key inflammatory pathways, reduce oxidative stress by activating Nrf2-mediated antioxidant responses, and regulate macrophage activity by suppressing pro-inflammatory cytokines like TNF-α, IL-1β, and IL-6 [52]. Additionally, algae-derived compounds exhibit antimicrobial properties. By inhibiting pro-inflammatory cytokines and enhancing antioxidant defenses, microalgae-derived bioactive compounds can support intestinal health and overall well-being [11,53]. These multifactorial benefits position microalgae as promising candidates in IBD management.

Beyond their nutritional and therapeutic value, microalgae offer environmental advantages. Their cultivation does not require arable land, and they can thrive in diverse aquatic environments, including saline and wastewater, thus conserving freshwater resources [54]. They also contribute to carbon dioxide mitigation through photosynthesis, making them an eco-friendly choice for sustainable food and feed production [47].

Despite the advantages of microalgae, there are ongoing challenges in scaling up production, extraction, and enhancing the digestibility and bioavailability of the nutrients they provide [55]. These challenges create a bottleneck for making microalgae-based products more cost-effective, scalable, and accessible for widespread use [55]. Overcoming these barriers is essential for transitioning from preclinical promise to practical application in IBD care.

**Antioxidant properties of microalgae.** Microalgae-derived antioxidants such as phycocyanin from *Spirulina* and carotenoids from *Chlorella* offer a dual benefit: ROS scavenging and inflammation modulation [49,56]. Recent in vivo models provide evidence for these protective effects. A study by Lu et al. (2020) [51] investigated the antioxidant activity of phycocyanin in radiation-induced intestinal toxicity in mice. Phycocyanin significantly increased the activities of SOD and GPx while reducing MDA levels in colonic tissues. These effects corresponded with enhanced intestinal barrier function and reduced inflammatory infiltration [51]. Comparative studies, such as those by Xie et al. (2019) [57], revealed that natural antioxidants from microalgae were more effective than synthetic alternatives in mitigating oxidative damage, as they simultaneously modulate inflammatory pathways like Toll-Like Receptor 4 (TLR4)/NF-κB while improving ROS detoxification mechanisms [51]. Specifically, Xie et al.’s study demonstrated that phycocyanin, a natural antioxidant, modulated the intestinal microbiota composition, enhancing bacterial diversity and richness. After phycocyanin intervention, there was a significant increase in bacterial abundance and diversity. The abundance of the saccharolytic bacteria families *Lachnospiraceae* and *Ruminococcaceae*, which produce butyric acid, increased, indicating improved gut health and barrier function. The study also observed a significant reduction in intestinal permeability and an increase in intestinal barrier function, as demonstrated by the decreased serum lipopolysaccharide levels by 27% and a substantial improvement in histological markers of intestinal health [57]. Together, these findings show that phycocyanin simultaneously targets oxidative stress and microbiome dysbiosis.

Another study by Shandily et al. (2022) [56] evaluated the carotenoid content in *Chlorella vulgaris* and demonstrated its ability to inhibit lipid peroxidation and scavenge ROS in oxidative stress-induced cellular models, suggesting its potential for therapeutic application in IBD [56].

**Prebiotic properties of microalgae and their effect on the microbiome.** Microalgae polysaccharides serve as prebiotic substrates for gut microbiota, promoting the growth of beneficial bacteria and the production of SCFAs, such as butyrate, acetate, and propionate, which play critical roles in maintaining gut health. Zhou et al. (2023) [48] demonstrated that *Spirulina* and *Chlorella* polysaccharides significantly enhanced the growth of *Lactobacillus* and *Ruminococcaceae* during in vitro colonic fermentation. This modulation of gut microbiota was associated with reduced markers of intestinal permeability, such as endotoxin and D-lactate, and improved epithelial barrier integrity [48].

Omar et al. (2022) [49] used a DSS-induced colitis model to further confirm these findings, showing that microalgae polysaccharides decreased gut dysbiosis by increasing the relative abundance of SCFA-producing bacteria. These effects were accompanied by a reduction in oxidative stress markers, such as ROS and lipid peroxidation products, thereby supporting gut barrier restoration and reducing inflammation [49].

Microalgae-derived polysaccharides selectively enhance the growth of beneficial gut bacteria, supporting their role in reducing inflammation. This symbiotic relationship promotes a balanced gut microbiome, essential for managing IBD. In another study by Yan et al. (2024) [58] researchers highlighted the therapeutic potential of *Chlorella pyrenoidosa* (CP) in treating UC. Using a DSS-induced colitis mouse model, CP significantly alleviated UC symptoms, including weight loss, colon shortening, and colonic tissue damage, while improving mucosal integrity and reducing inflammatory cell infiltration. CP reduced inflammatory markers such as TNF-α, IL-6, and IL-1β, and restored the expression of mucin (MUC2) and ZO-1, critical for maintaining intestinal barrier function.

It corrected gut microbiota imbalances by enhancing the abundance of beneficial bacteria (e.g., *Akkermansia*, *Alistipes*) and reducing harmful species (e.g., *Helicobacter*, *Staphylococcus*). CP also increased the production of SCFAs, particularly butyric acid, which was pivotal in suppressing colonic cell apoptosis and promoting intestinal health. Furthermore, CP modulated metabolic pathways linked to inflammation and apoptosis, improving overall colonic function. These findings establish CP as a promising nutraceutical or prebiotic for UC management, with butyric acid identified as a critical mediator of its therapeutic effects [58].

**Microalgae prebiotics and microbial resilience.** The study by Paterson et al. (2025) [59] explores the impact of *Nannochloropsis gaditana* (NG) on human gut microbiota and SCFA production using simulated gastrointestinal digestion and colonic fermentation. NG digests were found to significantly increase beneficial bacterial genera, such as *Akkermansia*, *Butyricicoccus*, *Lachnoclostridium*, *Eisenbergiella*, and *Marvinbryantia*, compared to inulin as a control. These changes contributed to higher levels of major SCFAs like butyric and propionic acids, with total SCFA production reaching 46.84 mM at 72 h, surpassing inulin. NG also promoted the production of minor SCFAs, such as valeric acid, known for its anti-inflammatory and immunomodulatory properties. Additionally, NG digests reduced harmful microbial populations, likely through competition and SCFA-mediated lowering of colonic pH [59]. This dual role highlights the delicate balance between SCFA production and its protective effects and the critical need to manage oxidative stress and inflammation to preserve gut health.

A stable gut microbiome reduces susceptibility to inflammation, thereby providing lasting relief for IBD patients and reducing IBD relapse, supporting long-term gut health [53]. A study by Stiefvatter et al. (2022) [60] highlights the potential of the microalga *Phaeodactylum tricornutum* (PT) as a dietary supplement to enhance gut health through its effects on the microbiota and SCFA production. In preclinical trials, PT-rich diets, formulated to emphasize either eicosapentaenoic acid (EPA) and fucoxanthin (Fx) or chrysolaminarin (Chrl), significantly boosted SCFA levels, particularly acetate and propionate, with some formulations increasing butyrate production. These improvements were linked to shifts in gut microbiota, including a reduced Firmicutes-to-Bacteroidota (F/B) ratio and increased abundance of beneficial bacteria like *Akkermansia* and *Clostridia vadin* BB60. Nutritional safety was confirmed, with high doses of PT components showing no adverse effects on intestinal permeability or inflammation while enhancing the absorption of EPA and reducing the liver’s n-6:n-3 fatty acid ratio. PT diets also preserved intestinal barrier integrity, as evidenced by stable levels of tight junction proteins such as ZO-1 and occludin. While an increase in TNF-α levels was noted under certain conditions, the overall findings suggest anti-inflammatory effects, likely mediated by EPA and Fx. These results underscore PT’s promise as a sustainable dietary intervention for improving gut health and promoting microbial balance [60].

Similarly, research by Wang et al. (2022) [61] underscores the therapeutic role of *Spirulina* platensis aqueous extracts (SP) in addressing UC. The study reveals that SP not only reduces inflammation and oxidative stress but also strengthens the intestinal barrier by enhancing the expression of tight junction proteins like ZO-1 and occludin. SP also modulates gut microbiota, increasing the abundance of beneficial bacteria such as *Lactobacillus* and *Akkermansia* while mitigating harmful species. This dual action of SP in reducing mucosal damage and restoring microbial balance reinforces its potential as a safe and effective adjunct therapy for IBD. Together, these studies highlight microalgae’s versatile and promising applications in gastrointestinal health management [61]. Building on this, recent studies have explored how microalgae may act synergistically with probiotics to amplify these benefits.

Another study by Xie et al. (2019) [57] assessed the effects of phycocyanin supplementation on gut microbiota composition in mice. The research employed six-week-old male mice divided into three groups: a control group, a low-dose phycocyanin group (50 mg/kg), and a high-dose phycocyanin group (100 mg/kg). Phycocyanin was administered daily by oral gavage over 28 days. The study utilized 16S rRNA sequencing to analyze microbial diversity and abundance changes. Significant findings included an increase in the relative abundance of butyrate-producing bacteria, such as *Lachnospiraceae* and *Ruminococcaceae*, which are essential for maintaining gut homeostasis and modulating inflammation through their production of SCFAs, maintaining intestinal barrier integrity, and reducing inflammation. Moreover, serum LPS levels, a marker of gut permeability, were significantly reduced in phycocyanin -treated groups. Histological analysis revealed improved villus height and goblet cell density in the ileum and colon, indicative of enhanced epithelial barrier function. The results demonstrated the dual role of phycocyanin in modulating gut microbiota and reinforcing intestinal barrier integrity, highlighting its potential as a therapeutic agent in IBD management [57].

## 6. Prebiotic Properties of Microalgae and Their Relation to Oxidative Stress

The synergistic potential of combining microalgal antioxidants with probiotic bacteria for gut health has been slowly recognized in recent studies [62]. Most of the studies that focus on algae and microorganisms’ synergetic effect tested the effect of adding dry algae powder to enhance the growth and composition of beneficial bacteria, mainly in the food and bioremediation industries [62,63]. Here, rather than focusing solely on the individual benefits of microalgae or probiotics, the following studies emphasize how their combination can enhance viability in both organisms, immune defence, and gastrointestinal balance.

Interestingly, not much research has been conducted on the synergistic effect of combining microalgae and probiotics to treat IBD. However, it is important to note that in addition to their prebiotic effects, microalgae directly influence gut health by fostering a symbiotic environment for probiotics. A study by Ferrer et al. (2024) [64] demonstrated that *Spirulina platensis* enhances the colonization and activity of probiotics such as *Bifidobacterium* by providing essential nutrients such as amino acids and polysaccharides. The research employed an in vitro intestinal epithelium model using Caco-2/TC7 cells treated with specific SCFAs derived from *Spirulina* polysaccharides. The experimental design included a simulation of oxidative stress induced by TNF-α at 5 ng/mL, followed by treatment with SCFAs at a physiological mixture ratio (60:25:15 for acetate–butyrate–propionate). Significant findings revealed a reduction in TNF-α-induced lipid peroxidation by over 50% and restoration of CAT and GPx activities to baseline levels. Additionally, SCFAs enhanced the production of SCFAs, reinforcing epithelial tight junction integrity and promoting a balanced gut microbiome [64]. It is important to note that while SCFAs such as butyrate, acetate, and propionate are key anti-inflammatory metabolites produced by gut bacteria, their efficacy can be impaired under oxidative stress and dysbiosis common in IBD [39,41]. Microalgae, by contrast, provide a multifaceted approach: in addition to serving as prebiotics that promote SCFA-producing bacteria [48,49], microalgae provide antioxidant compounds (e.g., phycocyanin, carotenoids) that scavenge ROS and reduce epithelial damage, thus restoring conditions necessary for effective SCFA metabolism [49,51]. Moreover, microalgae-derived bioactive compounds independently modulate immune responses by downregulating pro-inflammatory cytokines (e.g., TNF-α, IL-6) and upregulating antioxidant pathways (e.g., Nrf2), which SCFAs alone may not robustly achieve [21,22]. Therefore, microalgae are uniquely positioned to address both upstream causes (oxidative stress, immune dysregulation) and downstream effects (microbial imbalance and SCFA depletion), offering broader therapeutic coverage than SCFA supplementation alone [48,49].

## 7. Advancements in Algae-Based Therapeutic Delivery Systems

Recent studies have demonstrated the strong potential of microalgae for targeted drug delivery both in vitro and in vivo, as they can effectively load drug molecules through their active surfaces [65,66]. Oral drug delivery is the preferred and most commonly used route of drug administration for GI disease treatment, mainly owing to its safety, high patient compliance, convenience, and ease of production [67].

Zhong et al. (2021) [68] developed a microalgae-based oral drug delivery system for treating intestinal diseases using the helical-shaped cyanobacterium SP loaded with curcumin (SP@Curcumin). The researchers encapsulated curcumin within SP, evaluating its stability, drug-loading efficiency, and distribution within the gastrointestinal tract of mice. The formulation leverages SP’s natural properties to enhance the bioavailability and retention of curcumin while maintaining structural integrity through the stomach and effectively releasing the drug in the intestine. The main findings include SP@Curcumin’s capability to protect against oxidative stress and DNA damage in normal cells during radiation therapy, reduce proinflammatory cytokine production, and attenuate colon tissue damage in colitis models. The study confirms the dual therapeutic and protective effects of SP@Curcumin, highlighting its potential for treating both cancer and inflammatory intestinal diseases effectively [68].

In another study, Jester et al. (2022) [69] presents a comprehensive platform for producing and delivering protein therapeutics using genetically engineered SP as a scalable, food-grade biomanufacturing system. The researchers developed methods for stable chromosomal integration of therapeutic protein genes into SP using homologous recombination. Key features of the methodology include inducing natural competence in SP through co-culture with companion microbes, precise genetic insertion using flanking homology arms, and markerless engineering strategies. Expressed proteins—such as single-chain antibody fragments (VHHs)—achieved yields up to 29% of total soluble protein. The study demonstrated oral delivery of a *Spirulina*-based therapeutic containing an anti-*Campylobacter jejuni* VHH antibody (FlagV6-MBP), which showed potent efficacy in mouse infection models, reducing bacterial shedding by 3–4 logs and preventing disease symptoms. Importantly, the therapeutic proteins remained stable through gastric conditions when encapsulated in dried *Spirulina* biomass and were bioavailable in the intestine. The team also conducted a Phase 1 clinical trial with healthy volunteers, confirming safety, tolerability, and the absence of systemic absorption. This work establishes *Spirulina* as a low-cost, scalable, and orally bioavailable platform for protein biologics targeting enteric diseases and beyond [69].

Zhang et al. (2022) [70] further explored the use of SP as an oral carrier for amifostine (AMF), a clinically approved radioprotectant. Their work demonstrated that SP@AMF significantly enhanced the intestinal biodistribution of AMF compared to its free and capsule forms. The SP@AMF particles retained their structural integrity in gastric fluid and facilitated prolonged and uniform AMF release in the small intestine. Importantly, this system provided selective protection of intestinal tissues during radiation therapy without impairing tumor regression, preserved gut microbiota homeostasis, and prolonged survival in animal models [70].

Another notable advancement involves the fabrication of algae-based nanoparticles (aNPs) for oral delivery. Drori et al. (2024) [71] developed biomimetic nanoparticles derived from 14 types of edible algae. Among them, *Arthrospira platensis* (*Spirulina*) yielded nanoparticles with optimal characteristics: smallest size (126 ± 2 nm), highest mucoadhesive force, and most negative zeta potential (−38 mV), indicating strong interaction potential with the intestinal mucosa. These aNPs showed superior cellular uptake in Caco-2 cells and sustained drug release profiles, validating their utility as bioadhesive drug carriers for enhancing intestinal residence time and bioavailability [71].

In parallel, nature-engineered diatom biosilica platforms are gaining attention for their unique hierarchical porosity, ease of functionalization, and biocompatibility. Uthappa et al. (2018) [72] reviewed diatom species such as *Thalassiosira pseudonana* and *Coscinodiscus wailesii*, which feature uniform nano- to microscale pores ideal for high-efficiency drug loading and controlled release. Surface modification strategies—such as silanization and polymer coating—allow precise tuning of drug release profiles and targeting. These diatom-based systems are cost-effective alternatives to synthetic mesoporous silica and demonstrate potential across various delivery applications, including oral, dermal, and injectable routes [72]. The remarkable versatility, biocompatibility, and functional tunability of algae-based systems position them as a transformative platform for next-generation therapeutic delivery, particularly in gastrointestinal and inflammatory disease contexts.

A significant example of such synergy was observed in a study by Cantú-Bernal et al. (2020) [73]. The study investigated the potential synergy between the microalga *Chlorella sorokiniana* and probiotic strains *Bifidobacterium longum* and *Lactobacillus plantarum* in a dairy-based food matrix. The microalga was incorporated into a dairy dessert (flan) along with the probiotics. The study measured probiotic viability during refrigerated storage and evaluated antiviral activity against rotavirus using HT-29 intestinal cells. Results showed that *C. sorokiniana* significantly enhanced the shelf-life of *L. plantarum*, maintaining viable counts above the therapeutic threshold for up to 18 days, and extended viability beyond 34 days when both probiotic strains were co-inoculated with the microalga. Moreover, when rotavirus-infected HT-29 cells were treated with probiotic metabolites in combination with *C. sorokiniana*, viral infectivity was dramatically reduced to as low as 5%, compared to untreated controls. Notably, the microalga alone also exhibited strong antiviral properties. These findings indicate a clear synergistic relationship, where *C. sorokiniana* supports probiotic viability and amplifies their antiviral efficacy, suggesting its promising application in functional food formulations aimed at gut and immune health [73].

Microalgae-based antioxidant properties create a favorable oxidative environment that enhances probiotic survival and activity [74]. A study by Huang et al. (2024) [75] introduces a novel probiotic delivery system using a bacteria–microalgae symbiosis model, with SP as a natural carrier for *Escherichia coli Nissle* 1917 (EcN), designed to enhance the efficacy of treating IBD. This system, termed EcN-SP, demonstrates multiple advantages, including improved survival and intestinal colonization of probiotics by protecting EcN from the acidic gastric environment and digestive enzymes. The symbiotic relationship between SP and EcN significantly reduces inflammatory markers such as TNF-α and IL-6, alleviates weight loss and colon shortening symptoms, and preserves intestinal epithelial integrity in a DSS-induced colitis mouse model. The EcN-SP system restores gut microbiota diversity and balances bacterial communities disrupted by IBD, increasing beneficial bacteria like *Lactobacillus* and *Bifidobacterium* while reducing harmful bacteria such as Bacteroides. It also normalizes the Bacteroidetes/Firmicutes ratio, a key IBD marker. The SP carrier promotes EcN proliferation under various conditions, with effective release and colonization in the intestine facilitated by pH-dependent binding. Finally, long-term safety assessments reveal no adverse effects on major organs or blood parameters, confirming its biosafety for therapeutic use [75].

In their 2024 study [76], Han et al. present an innovative approach to enhancing probiotic therapy for intestinal disorders by developing a microalgae-assisted delivery platform known as SP@BC [76]. The core of this system involves the use of SP as a biocompatible carrier for oral probiotic delivery. The researchers coated the probiotic strain EcN with chitosan (forming BCCS) and anchored it onto the helical surface of SP through electrostatic self-assembly. This structure was designed to overcome key limitations of conventional probiotic therapies, such as low survival through the gastrointestinal tract and poor mucosal adhesion. The methodology combined physical and biochemical advantages: chitosan enhanced bacterial viability by shielding against acidic and oxidative environments, while the helical shape and size of SP promoted intestinal retention by becoming physically entrapped within intestinal villi. The antioxidative properties of SP, particularly its production of enzymes like superoxide dismutase, further contributed to preserving probiotic activity in inflamed intestinal conditions. In vivo evaluation using a murine model of colitis demonstrated that SP@BC administration significantly improved clinical symptoms. It enhanced intestinal barrier integrity, attenuated inflammation, and supported the restoration of microbial balance within the gut. The study underscores the simplicity, scalability, and safety of the SP@BC system, as both SP and chitosan are widely regarded as safe and suitable for clinical translation [76].

These findings highlight how microalgae can act as more than just prebiotics. The antioxidant and bioactive compounds found in species like SP appear to directly support probiotic activity and enhance their health-promoting functions in the host organism. The use of such co-culture systems not only improves probiotic viability and function but also introduces new bioactivities, such as antiviral protection and microbiome modulation, which are not as pronounced when either component is used alone.

As research in this area expands, such synergistic combinations hold promise for the development of next-generation functional foods and nutraceuticals aimed at improving gut health and systemic well-being.

## 8. Conclusions

Addressing IBD requires multi-targeted interventions that go beyond immune suppression to restore redox balance and microbial homeostasis. IBD is a multifaceted condition where oxidative stress and gut microbiota dysbiosis play central roles in pathogenesis and progression. Current therapies often fail to address these interconnected mechanisms, leaving a critical gap in comprehensive disease management. This review highlights the potential of microalgae as a holistic therapeutic strategy to overcome this gap, offering dual benefits through their potent antioxidant and prebiotic properties (Table 1 and Table 2).

**Figure 2:**
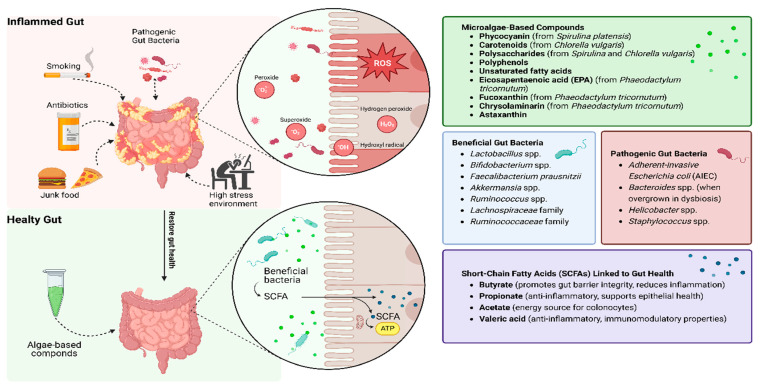
Microalgae-based compounds as a solution to repair oxidation stress in IBD patients. Microalgae compounds neutralize ROS, bolster antioxidant defenses, and promote beneficial gut bacteria, leading to SCFA production, improved barrier integrity, and reduced inflammation.

Microalgae-derived compounds such as phycocyanin and carotenoids effectively mitigate oxidative stress by neutralizing ROS and enhancing antioxidant defenses, as evidenced by their ability to restore intestinal barrier integrity and reduce inflammation in preclinical models [51]. Additionally, microalgae polysaccharides promote the growth of beneficial gut bacteria, including *Lactobacillus* and *Bifidobacterium*, while fostering the production of SCFAs such as butyrate, acetate, and propionate [48]. These metabolites are essential for maintaining gut health, reducing inflammation, and repairing epithelial damage (Figure 2). This dual role—supporting both host antioxidant capacity and microbial resilience—positions microalgae as a unique therapeutic class.

Emerging evidence also supports the synergistic potential of combining microalgae with probiotics. Such combinations leverage microalgae’s antioxidant and prebiotic effects to enhance probiotic viability and activity, providing a more robust restoration of gut microbial balance. Such combinations may lead to more durable remission outcomes in IBD. Advanced delivery systems, such as microalgae-based probiotic carriers, further enhance therapeutic efficacy while maintaining biosafety.

However, it is also important to acknowledge the possibility that microalgae-based interventions may not prove universally safe or effective in humans. Although well-known strains such as *Spirulina* and *Chlorella* are widely used in the aforementioned studies [48,49,58,61,69,73], the introduction of novel microalgal strains with high therapeutic potential requires rigorous evaluation [77]. According to European Food Safety Authority (EFSA) and Food and Drug Administration (FDA) standards [78], preclinical studies must be conducted to assess safety and efficacy, followed by clinical or nutritional intervention trials to establish therapeutic relevance in human populations. Moreover, certain strains may exhibit toxicity or engage in adverse interactions with concurrently prescribed medications, underscoring the necessity of thorough toxicological screening and drug interaction studies [77]. These safety concerns underscore the importance of carefully designed translational strategies before advancing microalgae-based interventions into routine clinical use [77].

Despite promising preclinical findings, challenges remain in translating these insights into clinical practice. Issues such as optimizing bioavailability, scale-up production, and long-term safety assessments must be addressed. Continued research and clinical trials are essential to establishing standardized protocols and formulations for using microalgae in IBD management. Continued research and development are essential for improving microalgae’s commercial viability and therapeutic applications, positioning them as significant contributors to the future of sustainable nutrition and health [4,34].

In conclusion, microalgae represent a sustainable and innovative approach to managing IBD by simultaneously addressing oxidative stress and gut dysbiosis. Their incorporation into treatment regimens, either as standalone therapies or as adjuncts to existing modalities, holds promise for improving patient outcomes and quality of life. By leveraging the bioactive potential of microalgae, a paradigm shift toward integrative and holistic care in IBD management appears within reach.

## Figures and Tables

**Table 1:** Summary table of methodology and main findingsfor In vitro studies.

Section	Species Studied	Compounds/Interventions	Methods	Results	References
Microalgae as a Natural Source in Treating IBD	In vitro colonic fermentation models; microalgae (*Spirulina*, *Chlorella*)	Pressurized liquid extracts (PLEs) from microalgae	In vitro colonic fermentation; NF–κB pathway assay; microbiota composition and SCFA measurement	Reduced NF–κB activation, increased beneficial bacteria, inhibited pathogens, enhanced SCFA production	Zhou et al. (2023) [48]
Microalgae (Antioxidant Properties)	Cellular models (oxidative stress-induced)	Carotenoids from *Chlorella vulgaris*	In vitro oxidative stress model; lipid peroxidation and ROS scavenging assays	Inhibited lipid peroxidation and effective ROS scavenging	Shandily et al. (2022) [56]
Microalgae (Prebiotic Properties and Oxidative Stress)	In vitro intestinal epithelium model (Caco–2/TC7 cells)	*Spirulina platensis* polysaccharides; SCFAs	In vitro model with TNF-α-induced oxidative stress; lipid peroxidation and antioxidant enzyme assays	A > 50% reduction in lipid peroxidation, restoration of CAT and GPx, enhanced epithelial integrity	Ferrer et al. (2024) [64]
Advancements in Algae-Based Therapeutic Delivery Systems	*Arthrospira platensis* (*Spirulina*)	Algae-based nanoparticles (aNPs)	Nanoparticle fabrication; size, mucoadhesive force, zeta potential measurement; cellular uptake and sustained drug release studies	Optimal nanoparticle size, high mucoadhesive force, sustained release for enhanced intestinal residence	Drori et al. (2024) [71]
Advancements in Algae-Based Therapeutic Delivery Systems	Diatom species (*Thalassiosira pseudonana*, *Coscinodiscus wailesii*)	Diatom biosilica platforms; surface modifications (silanization, polymer coating)	Literature review on drug loading and release profiles	Demonstrated potential for high-efficiency drug loading and controlled release in various applications	Uthappa et al. (2018) [72]
Microalgae Prebiotics and Microbial Resilience	Simulated human gut microbiota	*Nannochloropsis gaditana* digests	Simulated gastrointestinal digestion and colonic fermentation; SCFA measurement; microbiota analysis	Increased beneficial bacterial genera, elevated SCFA production, reduced harmful microbes	Paterson et al. (2025) [59]
Synergistic Effects of Microalgae and Probiotics	*Chlorella sorokiniana*; probiotic strains (*Bifidobacterium longum*, *Lactobacillus plantarum*)	*Chlorella sorokiniana* in a dairy-based matrix	Dairy dessert formulation; refrigerated storage viability tests; antiviral assays in HT-29 cells	Enhanced probiotic viability, extended shelf-life, dramatically reduced rotavirus infectivity (~5%)	Cantú-Bernal et al. (2020) [73]

**Table 2:** Summary table of methodology and main findings for In vivo studies.

Section	Species Studied	Compounds/Interventions	Methods	Results	References
Introduction	Crohn’s disease patients; gut microbiota	Oxidative stress-related genes (STAT3, MUC1, PRKAB1) and microbial metabolic pathways	Multi-omics Mendelian randomization study	Identified key genes regulating epithelial barrier integrity and links between oxidative stress and microbial dysbiosis	Xu et al. (2023) [6]
Mechanisms of Oxidative Stress in IBD and Gut Dysfunction	Murine colitis model (mice)	cDFPW1 polysaccharide from *Dendrobium fimbriatum*	DSS-induced colitis; histopathological analysis; tight junction protein immunohistochemistry; intestinal permeability assays	Upregulation of occludin and ZO-1, reduced serum biomarkers, activation of Nrf2, inhibition of NF-κB	Wang et al. (2022) [21]
Mechanisms of Oxidative Stress in IBD and Gut Dysfunction	Nrf2-deficient mice in DSS-induced colitis	Assessment of antioxidant enzyme deficiency	DSS-induced colitis; measurement of MDA levels and GPx activity	Increased colitis severity, elevated MDA, reduced GPx activity	Liu et al. (2022) [23]
Mechanisms of Oxidative Stress in IBD and Gut Dysfunction	Murine colitis model	Phytochemicals (curcumin, resveratrol)	DSS-induced colitis; measurement of SOD, CAT, inflammatory cytokines, and tight junction proteins	Reduced oxidative stress, increased antioxidant enzyme activities, decreased IL-1β and IL-6, enhanced occludin and ZO-1	Sahoo et al. (2023) [24]
Microalgae as a Natural Source in Treating IBD	Murine colitis model	*Chlorella vulgaris* extract	DSS-induced colitis; histological analysis; cytokine assays	Restored gut microbiota diversity, reduced epithelial damage, downregulated TNF-α and IL-6	Omar et al. (2022) [49]
Microalgae (Antioxidant Properties)	Mice (radiation-induced intestinal toxicity model)	Phycocyanin from *Spirulina*	Radiation-induced intestinal toxicity model; measurement of SOD, GSH-Px, MDA; intestinal barrier assessment	Increased SOD and GSH-Px, reduced MDA, improved intestinal barrier function	Lu et al. (2020) [51]
Microalgae (Prebiotic Properties)	Six-week-old male mice (DSS-induced colitis model)	Phycocyanin supplementation (low/high dose)	Oral gavage over 28 days; 16S rRNA sequencing; histological analysis; serum LPS measurement	Increased butyrate-producing bacteria, improved villus height and goblet cell density, reduced serum LPS, enhanced barrier function	Xie et al. (2019) [57]
Advancements in Algae-Based Therapeutic Delivery Systems	Mice; *Spirulina platensis* (cyanobacterium)	SP@Curcumin (curcumin-loaded *Spirulina*)	Encapsulation; stability and drug-loading efficiency assessment; in vivo distribution in mice	Protected curcumin through gastric passage, enhanced bioavailability, reduced oxidative stress and inflammation, attenuated colon damage	Zhong et al. (2021) [68]
Advancements in Algae-Based Therapeutic Delivery Systems	*Spirulina platensis*; protein therapeutics	Genetically engineered *Spirulina* producing anti-*Campylobacter jejuni* VHH antibody	Stable chromosomal integration; oral delivery in mice; Phase 1 clinical trial in healthy volunteers	High yield of therapeutic proteins, reduced bacterial shedding, stability in gastric conditions, safe in humans	Jester et al. (2022) [69]
Advancements in Algae-Based Therapeutic Delivery Systems	Animal models; *Spirulina platensis*	SP@AMF (amifostine-loaded *Spirulina*)	In vitro and in vivo evaluation of intestinal biodistribution and drug release	Enhanced intestinal biodistribution, prolonged uniform release, selective intestinal protection, preserved microbiota, prolonged survival	Zhang et al. (2022) [70]
Microalgae Prebiotics and Microbial Resilience	Murine colitis model (UC)	*Spirulina platensis* aqueous extracts (SP)	DSS-induced colitis; measurement of tight junction proteins, inflammatory cytokines, gut microbiota analysis	Reduced inflammation and oxidative stress, strengthened intestinal barrier, increased beneficial bacteria	Wang et al. (2022) [61]
Microalgae Prebiotics and Microbial Resilience	*Phaeodactylum tricornutum* (PT)	PT-rich diets (EPA, fucoxanthin, chrysolaminarin)	Preclinical animal trials; measurement of SCFA levels, gut microbiota, tight junction proteins, inflammatory markers	Boosted SCFA levels, reduced Firmicutes/Bacteroidota ratio, increased beneficial bacteria, preserved barrier integrity, anti-inflammatory effects	Stiefvatter et al. (2022) [60]
Synergistic Effects of Microalgae and Probiotics	*Spirulina platensis*; *Escherichia coli Nissle* 1917 (EcN)	EcN–SP symbiotic delivery system	In vitro and in vivo (DSS-induced colitis model) experiments; measurement of inflammatory markers, microbiota composition, intestinal colonization	Improved EcN survival and colonization, reduced inflammatory markers, restored epithelial integrity, balanced gut microbiota	Huang et al. (2024) [75]
Synergistic Effects of Microalgae and Probiotics	*Spirulina platensis*; *Escherichia coli Nissle* 1917 (EcN)	SP@BC (*Spirulina* with chitosan-coated EcN)	Chitosan coating; electrostatic self-assembly; in vivo murine colitis model	Enhanced probiotic viability, improved intestinal barrier integrity, reduced inflammation, restored microbial balance	Han et al. (2024) [76]
